# Biological effects of negative air ions on human health and integrated multiomics to identify biomarkers: a literature review

**DOI:** 10.1007/s11356-023-27133-8

**Published:** 2023-05-12

**Authors:** Sha Xiao, Tianjing Wei, Jindong Ding Petersen, Jing Zhou, Xiaobo Lu

**Affiliations:** 1 International School of Public Health and One Health, Heinz Mehlhorn Academician Workstation, Hainan Medical University, Haikou, 571199 China; 2 Department of Toxicology, School of Public Health, China Medical University, Shenyang, 110122 China

**Keywords:** Negative air ions, Temporal and spatial dynamics, Health effect, Anti-inflammation, Omics

## Abstract

Environmental pollution seriously affects human health. The concentration of negative air ions (NAIs), which were discovered at the end of the nineteenth century, is one of the factors used to evaluate air quality. Additionally, NAIs have been widely considered markers by scholars due to their unique biological function. The aim of this study was to summarize existing research and propose future research on the generation and temporal and spatial dynamic patterns of NAIs concentrations as well as the relationship between NAIs and human health. We identified 187 studies (published January 2013–January 2023) that met our inclusion criteria. Fourteen English studies evaluated the effects of NAIs on depression, the cardiovascular system, the respiratory system, reproduction and development, cognition, and sports muscle injury. Only two studies reported the associations of NAIs exposure with metabolic omics. NAIs concentrations vary temporally with solar radiation, air temperature, and relative humidity, while the temporal dynamic patterns of NAIs are affected by season, time, meteorological factors, air quality index, geographical location, forest vegetation, and other factors. Researchers have shown that exposure to NAIs may benefit our health by changing amino acid metabolism, which mainly manifests as increased anti-inflammation and reduced inflammation and antioxidation. Furthermore, exposure to NAIs promotes energy production, affects the expression of c-fos, and regulates 5-HT levels. There has been considerable interest in the potential effects of NAIs on human health and well-being, but the conclusions have been inconsistent and the mechanisms remain unclear. The use of omics to elucidate the biological mechanism of NAIs is relatively new and has some advantages.

**Graphical Abstract:**

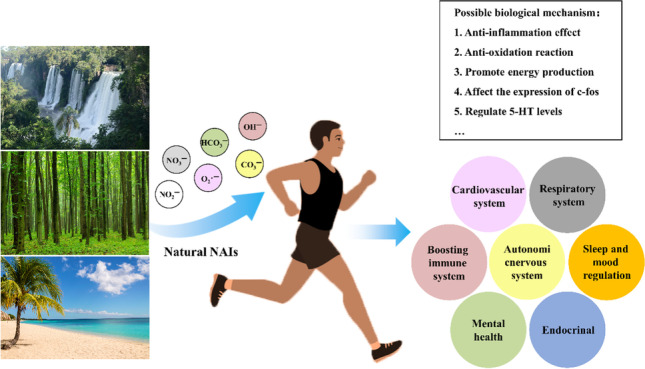

## Introduction

With the accelerating process of industrialization and urbanization, the problem of environmental pollution has gradually become more prominent, thereby seriously threatening the quality of human life and physical and mental health. Therefore, it is important to pay more attention to the living environment. Furthermore, since people spend more than 80% of their time indoors, previous studies have indicated that indoor air pollution has an equal or more significant effect on human health compared with ambient air pollution (Jia X et al. 2018).

Recently, urban air quality has become a hot research topic around the world, and the concentration of negative air ions (NAIs) is regarded as an essential indicator of urban air quality and the effects of forests on human health (Liu S et al. 2022). NAIs are negatively charged gas ions that are formed and generated by sunlight, radiant or cosmic rays, plant-based sources of energy, or other natural and artificial energy sources, also known as colorless, tasteless, and small particle size (Nazaroff WW et al. 2020). NAIs can effectively reduce the concentration of particulate matter, especially PM_2.5_ and PM_10_ (Jiang SY et al. 2021), and prevent volatile organic compounds (VOCs) from binding to PM_2.5_ (Zhang C et al. 2020), thereby improving air quality. Additionally, NAIs can be found at high concentrations in the atmosphere of forests, waterfalls, beaches, and so on, up to 0.5 × 10^3^–10 × 10^3^ ions/cm^3^. The NAIs distribution has noticeable diurnal, monthly, and interannual changes related to meteorological factors such as solar radiation, air temperature, and relative humidity, among others.

Many epidemiological studies and clinical investigations have reported that NAIs have many potential biological effects such as lowering blood pressure, improving body immunity, and improving erythrocyte deformability (Iwama H et al. 2002). In addition, they have also been found to enhance metabolism (Iwama H 2004), affect emotions, and inhibit the viability of airborne gram-positive and gram-negative bacteria (Comini S et al. 2021; Bowers B et al. 2018). NAIs can directly stimulate the nerve reflex and the humoral system. They play a physiological regulative role (Lv J et al. 2011; Wu CC et al. 2006) or affect the composition and distribution of ions in blood by releasing electric charge to improve the content of blood oxygen. Furthermore, they enhance the absorption and utilization of blood oxygen to promote the redox reaction of the body (Sirota TV et al. 2008). The mechanisms leading to NAIs-related biological effects are multifactorial and not yet fully understood. In recent years, high-throughput omics approaches have been utilized as powerful techniques to explore the effect of exposure to environmental factors on human physiology, thereby increasing knowledge regarding the biological response to exposure and the underlying molecular mechanism. However, their implications for air quality and health hazards have not been reviewed comprehensively.

The objective of this review was to evaluate the research literature published from 2013 to 2023 to assess the potential biological effects of NAIs on human health. We discussed the current state of the literature on the generation and dynamic patterns of atmospheric negative ions as well as the relationship between NAIs and human health. We focused on publications that applied omics studies to the biological effects of NAIs, as these are relatively new methods and have implications for improving air quality and reducing health hazards.

## Literature search and study identification

**Fig. 1:**
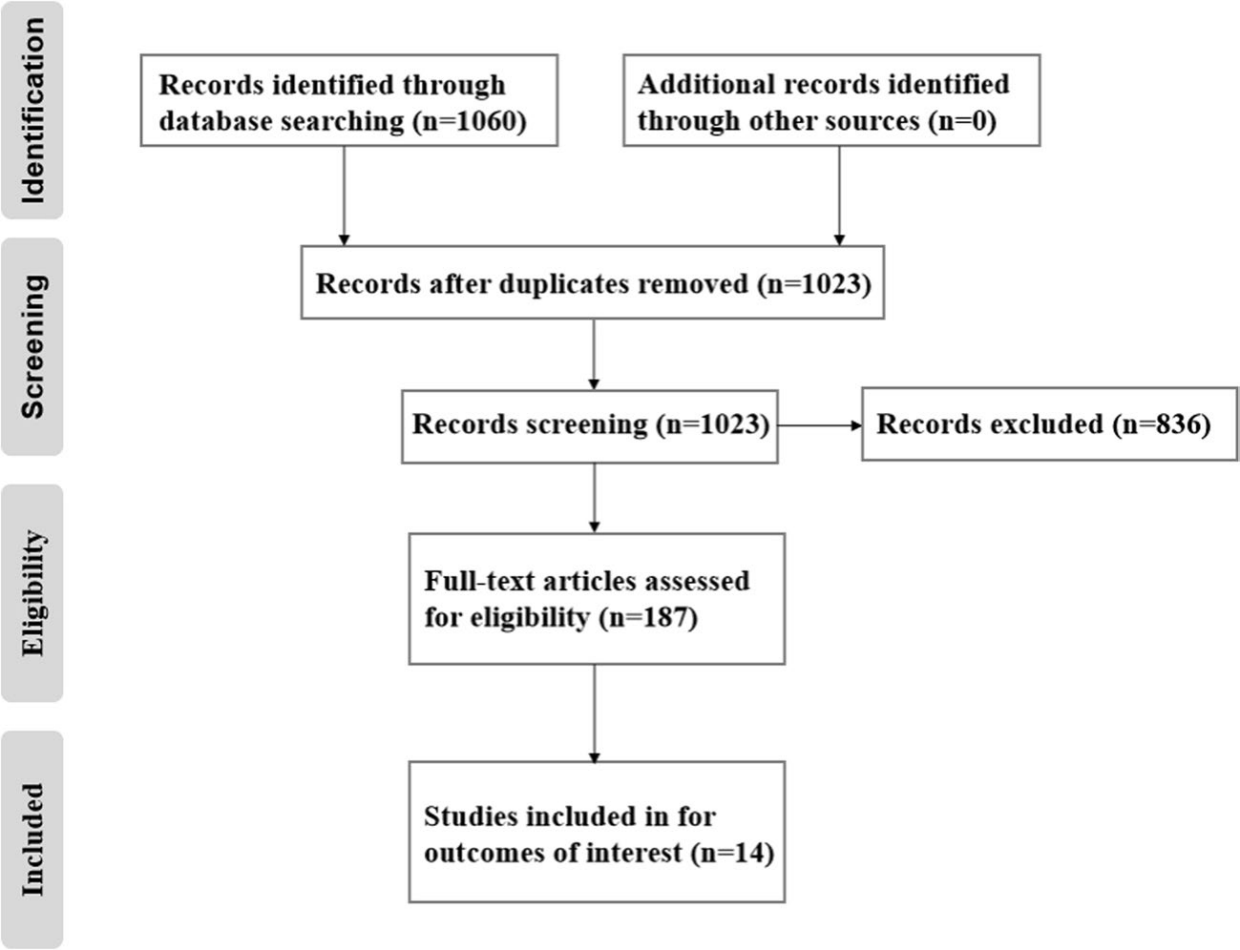
Flow diagram of the literature search and selection

This systematic review of studies on NAIs was initiated by searching the PubMed, Embase, and China National Knowledge Infrastructure databases to identify relevant experimental studies published between January 1, 2013 and January 1, 2023. Fig. 1 shows the step-by-step identification and selection process. Three keywords, i.e., “negative,” “air,” and “ion”, were used to search all collected articles in the databases. The database searches yielded a total of 1060 articles, and 675 could be retrieved in full. We then screened these articles by reviewing the titles and abstracts to exclude unrelated articles; a total of 187 references remained.

Identical search strings were used for the PubMed and Embase databases to obtain articles related to exposure (air ions, atmospheric ions, ionization, ionized air, and negative ions) and the outcomes of interest (depression, cardiovascular system, respiratory system, reproduction and development, cognition, and sports muscle injury). In addition, we manually reviewed the reference lists of all retrieved articles to identify potentially eligible publications. Ultimately, 14 English-language studies met our inclusion criteria and were included in the analysis (Table 1).

**Table 1:** The characteristics of the 11 eligible articles with their NAIs exposure level, study object, and findings

First author/year of publication	Study object	NAI exposure level	Findings
Olimpia P/2013^a^	Study 1. Ten male Wistar albino rats were divided into the control and experimental groups in random. Study 2. Twenty height clinically stable adult patients (age range 18–43 years) with persistent asthma were randomly allocated to the NAI treatment group or the placebo group (self-management of symptoms). Study 3. Thirty-two seasonal affective disorder (SAD) patients were randomized into four groups in a randomized controlled, parallel group clinical trial.	Study 1. Consequence of 2 weeks of 15 min NAIs exposure (the animals rested under constant temperature of 24 ± 1 °C and humidity of 47 ± 1%). Study 2. NAIs treatment (3 weeks of 25 min exposure). Study 3. Group 1: NAIs on/lamp on; group 2: NAIs off/lamp on; Group 3: NAIs on/lamp off, and control group received a placebo treatment (with NAIs device disabled). Treatments were taken in the early morning for 30 min over 21 days, followed by withdrawals.	Study 1. NAIs affect learning in a maze; the experimental group showed a significant reduction in error and time scores. Study 2. NAIs treatment (3 weeks of 25 min exposure) had improved scores on assessments of depression (Beck Depression Inventory, BCI) and anxiety (STAI-X) and led to a significant reduction in asthma outcomes. Study 3. NAIs with or without UV lamp both appear to act as specific antidepressants for men and women and appear to influence performance on cognitive tasks.
Ma M/2014^b^	Fifty-six hyperlipidemia patients were randomly divided into an observation group and a control group.	The patients in the control group engaged in tai chi exercise for approximately 60 min once a day; the patients in the observation group, in addition to performing tai chi exercise, were treated by inhalation of 10^3^ ions/cm^3^ NAIs.	Tai chi exercise combined with inhalation of NAIs can yield better effects on blood lipid indicators than tai chi exercise alone.
Nimmerichter A/2014^bc^	Fourteen male athletes were randomly divided into two groups in a double-blind study.	Exposure to 0.1 ± 0.06 × 10^3^ and 220 ± 30 × 10^3^ ions/cm^3^ NAIs 20 min after exercise.	NAIs exposure had no effect on the levels of epinephrine, norepinephrine, blood lactic acid, and aerobic metabolism.
Yamamoto D/2014^d^	Twenty female pregnant SD rats were randomly divided into two groups.	0, 7.0 × 10^6^ ions/cm^3^ NAIs, 6 h/day, 20 consecutive days.	NAIs had no effects on body weight, food consumption, and clinical signs; they also had no effects on animal embryo fetal development and foetal morphology.
Wallner P/2015^bce^	Twenty healthy adults, half were male and half were female, were randomly divided into two groups.	1038 ions/cm^3^, 2194 ions/cm^3^ NAIs, 2 h	NAIs exposure can increase sympathetic activity and improve cognitive ability. They had no effect on pulmonary function.
Yamamoto D/2015^e^	F0: ninety-six SD rats, half were male and half were female, were randomly divided into two groups (24 males and 24 females respectively), and mating was carried out between the same treatment groups. F1: forty-one offspring rats (21 males and 20 females) were born in the control group, and 46 offspring rats (23 males and 23 females) were born in the NAI exposure group. F0 and F1 are still the same processing group.	0, 7.0 × 10^6^ ions/cm^3^ NAIs, 6 h/day, 10 consecutive weeks.	The weight of the thyroid gland, pituitary gland, and uterus increased in parental rats. Except for the increase in food intake on the 20th day of pregnancy, NAIs had no effect on the first generation of offspring. They also had no effects on reproduction, development, and organ histology of the two generations.
Bowers B/2018^a^	Forty female patients with SAD were randomly divided into 4 groups in a single-blind study.	0, 2.0 × 10^6^ ions/cm^3^ NAIs, 30 min/day, 60 min/day, 18 consecutive days.	NAIs can alleviate depression and atypical SAD symptoms. The improvement among patients with morning sleep was greater than that among patients with night sleep.
Chu CH/2019^e^	Thirty-nine adults (28 males and 11 females) were randomly divided into two groups.	Exposure after working for 6 h, 26.13 ± 23.41 ions/cm^3^, 1 489.30 ± 148.92 ions/cm^3^ NAIs, 1 h.	NAIs may improve the basic information processing ability of volunteers by increasing neuronal activity.
Dong W/2019^bc^	Forty-four healthy middle school students (24 boys and 20 girls) were randomly divided into two groups.	12 997 ions/cm^3^ NAIs，10 h/day, 5 consecutive working days.	HRV showed a negative change, and the change in HRV was greater at high concentration of NAIs. FEV1 increased by 4.4% and FeNO decreased by 14.7%.
Ho CS/2020^f^	Thirty-eight badminton players (20 males and 18 females) were randomly divided into 4 groups in a double-blind study.	After exercise, 0, 300, 3000, and 30,000 ions/cm^3^ NAIs were given as adjuvant therapy.	The levels of TNF-a, creatine kinase and lactate dehydrogenase decreased significantly. NAIs alleviate the fatigue caused by muscle overload.
Liu S/2020^bc^	Forty-four healthy middle school students (24 boys and 20 girls) were randomly divided into two groups.	12,997 ions/cm^3^ NAIs，10 h/day, 5 consecutive working days.	The increase in NAIs can improve respiratory function mainly through promoting energy metabolism and anti-inflammatory and antioxidant pathways. The increase in NAIs can reduce HRV mainly by reducing energy metabolism and antioxidant pathways.
Cheng YH/2022^b^	Forty-five female Wistar rats were randomly divided into four groups.	Control group, LPS treated group, control group + NAIs-treated group, and LPS treatment + NAIs-treated group. The NAIs treatment of the rats by 10 cm distance of NAIs exposure was performed for 210 min.	NAIs have antioxidant and anti-inflammatory effects and can promote wound healing.
Hu YQ/2022^a^	Six-week-old male C57BL/6 mice were randomly divided into four groups.	(1) Control mice, (2) chronic mild stress (CMS)-treated mice, (3) control mice with 4.0 × 10^4^ ions/cm^3^ NAIs exposure, and (4) CMS-treated mice with 4.0 × 10^4^ ions/cm^3^ NAIs exposure for 30 days.	NAIs intervention is able to ameliorate CMS-induced depression-like behaviors in mice.
Liu S/2022^b^	Thirty-one healthy adults were randomly divided into four groups in a repeated-measure panel study.	The participants stayed in the forest for 3 days and 2 nights on different dates. After entering the forest, they were required to stay in the forest environment for 50 h. NAIs exposure was 68.11 (138.20) ions/cm^3.^	Forest NAIs can improve cardiac autonomic nervous function.

^a^ Depression. ^b^ Cardiovascular system. ^c^ Respiratory system. ^d^ Reproduction and development. ^e^ Cognition. ^f^ Sports muscle injury

## Overview of NAIs

### NAIs and their generation

Neutral gas molecules in the atmosphere combine with free electrons to form negatively charged atmospheric negative ions, which is the general name of negatively charged particles in the atmosphere. For example, atmospheric negative oxygen ions refer to the negatively charged oxygen ions formed by the combination of oxygen molecules and free electrons in the atmosphere. They are colorless and tasteless and are also known as small particle-size negative ions, accounting for approximately 10–20% of the content of atmospheric negative ions (Gui HL et al. 2018).

**Fig. 2:**
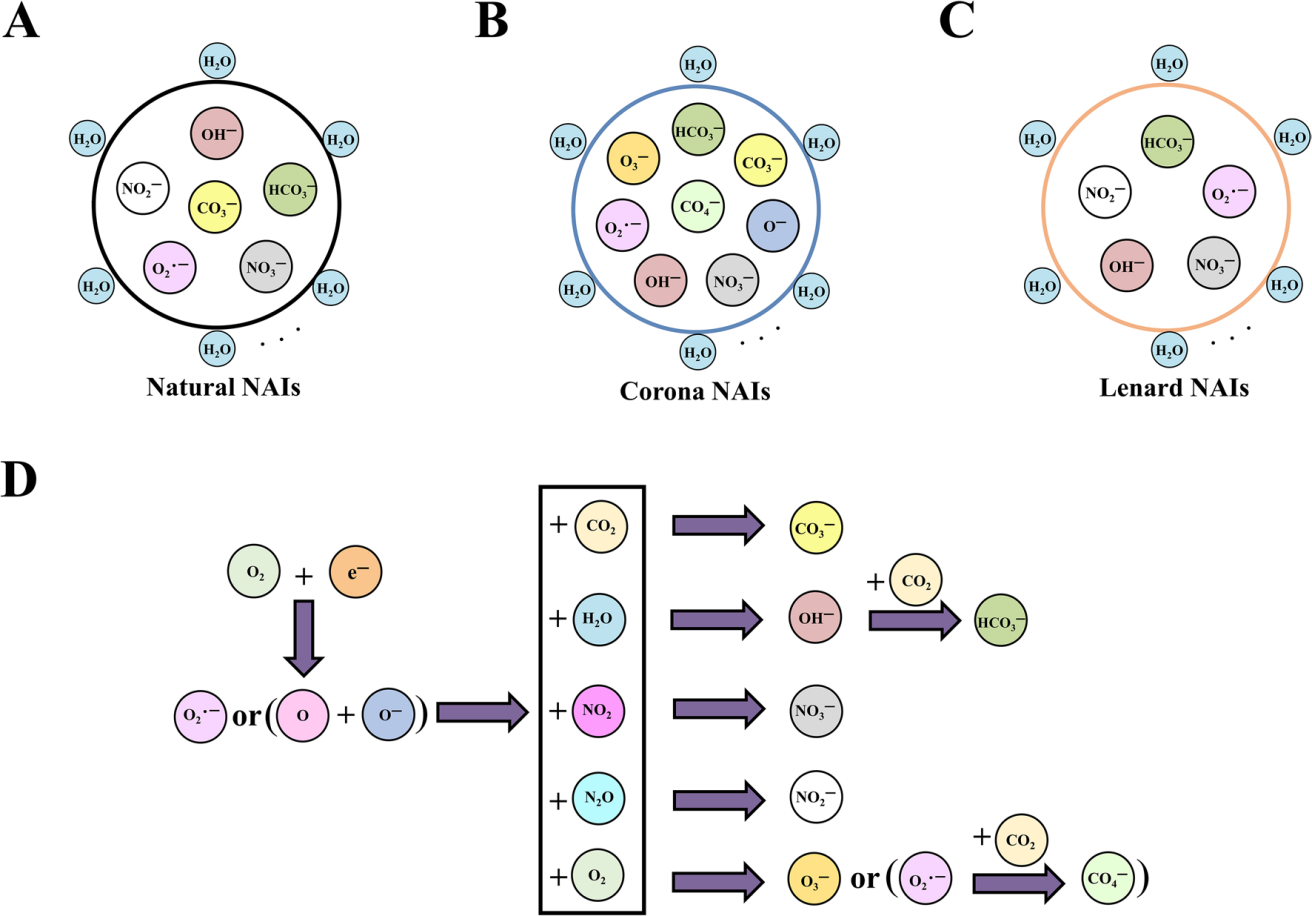
NAIs compositions from different generation sources. **A** Natural NAIs. References: Goldstein N 2002; Lin HF et al. 2017. **B** Coronavirus NAIs. Reference: Sekimoto K et al*.* 2013. **C** Lenard NAIs. Reference: Parts TE et al. 2007. **D** The evolution of oxygen-based NAIs. Reference: Lin HF et al. 2017

There are three primary sources of atmospheric negative ions. ① Under the action of cosmic rays, ultraviolet rays, trace element radiation, high-voltage electric fields, lightning, and water molecule collisions, electrons ionize and escape into the air, becoming free electrons with a negative charge, which are then captured by air molecules, aerosols, and fine particles, and negatively charged. ② In the environment of waterfalls, waves, and rainstorms, water mist with a negative charge is formed, which is taken away by air flow. ③ Plant “tip discharge” and photosynthesis release free electrons and combine with oxygen or water molecules to form atmospheric negative ions (LI G F et al. 2019a, 2019b). NAIs components produced by various sources of NAIs, and the evolution of oxygen-based NAIs are shown in Fig. 2 (Jiang SY et al. 2018).

### Temporal and spatial dynamic patterns of NAIs concentration

There are diurnal and seasonal changes in NAIs concentration. Most Chinese studies show that the concentration of NAIs in the air is higher in the morning and night, lower in the afternoon, higher in summer and autumn, and relatively lower in winter (Li A et al. 2022; Jiang ZH et al. 2021; Yao YP et al. 2019; Zhu SHX et al. 2019; LI Q Y et al. 2019a, 2019b; Zhang Yong et al. 2018). Furthermore, the concentration of NAIs is positively correlated with altitude (Zhang Yong et al. 2018; Jin Q et al. 2015). Li A et al. (Li A et al. 2022) found that the daily dynamics of NAIs concentration showed a bimodal curve in the Chinese Academy of Forestry (Fuyang District, Hangzhou, China, 30°03′35″N, 119°57′7″E). The peak NAIs concentration usually occurred in the early morning (5:00–7:00) and afternoon (15:00–17:00), and the lowest concentration usually occurred around noon (11:00–13:00). On a monthly scale, NAIs concentration was relatively high in February and August and relatively low in May and December. On the seasonal scale, NAIs concentration was significantly higher in summer than in other seasons. Autumn had the second highest NAIs concentration. Another study analyzed the continuous monitoring data of NAIs at three monitoring stations in Shennongjia, Yichang, and Jingzhou in Hubei Province. The results concluded that the concentration of NAIs was higher in summer and lower in winter, and that the concentration of NAIs was significantly higher at night than during the daytime. The highest value occurs between 4:00 and 7:00 in the morning (Gui HL et al. 2018). Yao YP (Yao YP et al. 2019) found that NAIs concentration has significant daily and monthly variation characteristics, with low concentration in the afternoon (13:00–16:00) and high concentration at night and in the morning (22:00–7:00). The concentration of NAIs was high from April to September and low in winter according to observations at 53 monitoring stations in Zhejiang Province in 2016.

It has been demonstrated that the concentration of NAIs varies in different observation areas. The concentration of NAIs in forest air is mostly between 900 and 5000/cm^3^ (Zhu SHX et al. 2019; LI Q Y et al. 2019a, 2019b; Zhang Y et al. 2018). The concentration in waterfalls and streams can be up to 10,000/cm^3^ (Wang W et al. 2013). Urban green space is 150–700/cm^3^ and varies with different green space compositions. The concentration of NAIs in urban traffic areas is generally between 200 and 300/cm^3^ (Yuan XY et al. 2014). Accordingly, the overall trend is arbor and shrub > arbor > shrub (Fei L et al. 2020).

### Effect of environmental factors on NAIs concentration

The concentration of NAIs correlates with meteorological factors such as air temperature (Ta), relative humidity (RH), wind and radiation, forest vegetation, and environmental conditions, but the research conclusions are different due to the influence of study area, season, and forest type. Li A et al. (Li A et al. 2022) showed that the air quality index (AQI) was a key factor affecting NAIs concentration compared to Ta and RH, especially PM and ozone; they also found that NAIs concentration had a negative correlation with these indicators and was significantly higher under favorable air quality conditions than under polluted air conditions. NAIs concentration and Ta showed marked piecewise characteristics, with NAIs increasing linearly with rising Ta only if the Ta was separated into three ranges of − 5 to 10 °C, 10 – 30 °C, and 30–40 °C. NAIs concentration was correlated with RH spanning all seasons, and water was an important factor affecting the distribution of NAIs concentration in different time series, as Li C et al. reported (Li C et al. 2021). Additionally, Peng LY et al. found that the NAIs concentration in different garden types has a differing correlation with Ta, RH, and precipitation, but that both are positively correlated with rainfall. The NAIs concentration of primitive broad-leaved forests has a negative correlation with Ta and a positive correlation with RH, while it is positively correlated with Ta and negatively correlated with noontime and RH in plantations (Peng LY et al. 2020). Other studies have reported the relationship among NAIs concentration, air oxygen content, wind speed, altitude, and precipitation (Zhu SHX et al. 2019; Zhang Y et al. 2018; Chen BH et al. 2019).

Most conclusions on the influencing factors of NAIs concentration mainly come from research on the influencing factors of NAIs contents in forest areas, while research on other types of NAIs concentration is relatively scarce. Therefore, Feng YM analyzed the influencing factors of NAIs concentration in the air of different habitats of an urban ecosystem, and the findings showed that the NAIs concentration in wetland parks and campuses was positively correlated with Ta, RH, and wind speed, and that the NAIs concentration in a square, an arboretum, and a residential area was positively correlated with Ta and RH (Feng YM et al. 2017).

## Evidence of the association between NAIs and their biological functions in human health

NAIs were first discovered in the natural environment at the end of the nineteenth century (Krueger AP 1972; Krueger AP et al. 1976). By the beginning of the twentieth century, NAIs were found to have a variety of potential biological functions including regulating respiratory system function, sedation, hypnosis, hypotension, regulating mood, neurological function, metabolism, and endocrine function, among others, in human health (Charry JM 1984; Day DB et al. 2018). Negative ion generators (NIAPs) have been widely utilized in various living environments and workplaces, and with the continuous development of science and technology, they have been gradually optimized, including the elimination of ozone as a byproduct (Jiang SY et al. 2021). Historically, various physiological or health effects related to exposure to charged air ions have been suggested, but the evidence of these effects is still ambiguous, and there seems to be a lack of in-depth mechanistic research.

### NAIs exposure and cardiovascular events

Previous studies have suggested that NAIs can effectively inhibit the sympathetic nerve and activate the parasympathetic nerve. Additionally, the paraventricular nucleus of the hypothalamus (PVN), locus coeruleus (LC), and nucleus ambiguous (NA) plays a key role in the regulation of sympathetic nervous system activity (Lucini D et al. 2020; Farrell MC et al. 2020). Therefore, it is speculated that NAIs can affect the autonomic regulatory activity of PVN, LC, and Na neurons, thus affecting autonomic nerve function to adjust heart rate and blood pressure. However, the neural mechanism of NAI-mediated autonomic regulation is unclear. Bailey WH et al. reviewed the literature on animal experimentation and the potential biological effects of NAIs published from 1935 to 2015 (Bailey WH et al. 2018). Suzuki S et al. (Suzuki S et al. 2008) preliminarily explored the mechanism of NAIs regulating the activity of the autonomic nervous system. The laboratory selected adult male Wistar rats and randomly divided them into two groups. One group was exposed to 5000–8000/cm^3^ NAIs air, and the other group was exposed to approximately 400–500/cm^3^ normal air to control temperature and humidity. Blood pressure (BP), heart rate (HR), and heart rate variability (HRV) were monitored. The expression of c-fos in the PVN, LC, Na, and nucleus tractus solitarius (NTS) was detected by immunohistochemistry. The results showed that NAIs significantly reduced blood pressure and heart rate and increased the high-frequency power of the HRV spectrum. The expression of c-fos was significantly downregulated in the PVN and LC regions and upregulated in the NA and NTS regions. After vagotomy, the above physiological changes and neuronal activity were not observed. NAIs can regulate autonomic regulation by inhibiting the activity of PVN and LC neurons and activating NA neurons, and the vagal nerve may mediate these effects.

Additionally, some animal studies have not found that positive or negative air ion exposure affects heart rate, respiratory rate, or blood pressure. In 2021, Kim M et al. (Kim M et al. 2021) published an in vitro study to explore the antioxidant and anti-inflammatory pathways of NAIs. After NAIs-exposed or unexposed HaCaT cells were treated with particulate matter (PM), the levels of reactive oxygen species (ROS) and the inflammatory factor IL-1 were detected. The expression levels of nuclear transcription factor activator protein 1 (AP1) and p38 protein were also detected at the same time. The results showed that NAIs could exert anti-inflammatory and antioxidant effects by inhibiting the ROS/p38 MAPK (mitogen-activated protein kinase)/AP1 pathway in HaCaT cells exposed to PM.

Several population investigations have investigated the health effects of NAIs exposure. Dong W et al. published a randomized, double-blind crossover test in 2019 (Dong W et al. 2019). In the study, 44 students in Beijing were selected to use commercial anion air purifiers for 5 weeks to monitor the indoor NAIs, PM, black carbon (BC), and ozone concentrations and observe the changes in HRV of volunteers. The results demonstrated that HRV exhibited a negative change, and that the alteration of HRV was more significant at a high concentration of NAIs. However, some studies (Liu S et al. 2020; Gui HL et al. 2018) have not found that NAIs exposure can improve cardiopulmonary function in healthy people. It is considered to be associated with the adverse effects of byproduct ozone, or the beneficial effect is only related to the decrease in PM_2.5_ concentration.

### Evidence on the function of NAIs in the respiratory system

High-concentration NAIs inhalation may improve lung function, regulate metabolism, and treat asthma symptoms. Alexander DD et al. (2013) systematically reviewed 23 studies related to negative ions and respiratory system function published from 1933 to 1993. The research population included in the literature involved infants, adolescents, and adults. The sample size varied from 8 to 123 subjects; most studies focused on 10–30 persons. There are few large-sample studies. A double-blind or single-blind crossover test was mainly used, and the exposure range of negative ions was 1600 to 1,500,000/cm^3^. Among them, two studies reported that NAIs exposure can improve the symptoms of patients with bronchial asthma. Overall, the review concluded that negative ion exposure did not significantly improve respiratory function or asthma symptoms. However, these studies are relatively old, limited by small sample sizes and different experimental methods, and had inconsistent conclusions. Dong W et al. (2019) also observed volunteers’ respiratory function changes and analyzed the correlation with environmental factors. The results showed that after using the air purifier, the PM_0.5_, PM_2.5_, PM_10_, and BC could be reduced by 48, 44, 34, and 50%, respectively. The concentration of NAIs increased from 12/cm^3^ to 12,997/cm^3^. The forced expiratory volume in 1 s (FEV1) of volunteers increased by 4.4%, and fractional nitric oxide (FeNO) decreased by 14.7%. Therefore, it could be considered that using a negative ion purifier can significantly improve the function of the respiratory system. Liu S et al. (2020) found that an increase in NAIs concentrations and a decrease in PM levels can improve respiratory system function by accelerating energy metabolism and improving anti-inflammatory and antioxidant capacity.

Other population studies have explored the effect of a high concentration of NAIs exposure on respiratory function during exercise or evaluated the effect of exercise and NAIs therapy on patients with respiratory diseases, but the research conclusions are also inconsistent (Su YF et al. 2018; Wen LY et al. 2017; A. Nimmerichter A et al. 2014; Mao QG et al. 2016; Shi YB et al. 2016). Su YF et al. (2018) evaluated the therapeutic effect of load-breathing training under NAIs synergistic treatment on 50 smokers with moderate and mild chronic obstructive pulmonary disease. The results showed that the volunteers exposed to high levels of NAIs exhibited a more significant improvement in lung function indices. Wen LY found that the level of NAIs is positively correlated with the training effect on respiratory function. Training in a high concentration of NAIs is conducive to improving respiratory muscle strength and pulmonary ventilation function (Wen LY et al. 2017). However, Nimmerichter A et al. did not find that a high concentration of NAIs (220 ± 30 × 10^3^/cm^3^) exposure had any effect on oxygen metabolism during exercise (Nimmerichter A et al. 2014).

Several researchers have discussed the effects of NAIs exposure on the respiratory system in experimental animals. Bailey WH et al. (2018) systematically reviewed the effects of air ions on the ciliary flow in the trachea of anaesthetized rabbits, rats, guinea pigs, and mice and their potential relationship with the level of neurotransmitter haemolysin. Negative ions can reduce ciliary activity and mucus flow in the trachea. However, there is a lack of sufficient data on the experimental design and results, including the lack of statistical analysis, the failure to control temperature, humidity, possible byproducts, and other factors, and the inability to quantitatively evaluate these effects. Other laboratories have reported that their studies have not replicated the above results. Sirota TV et al. reported that daily exposure of rats to NAIs with a concentration of 100,000–600,000 ions/cm^3^ produced by a Lustre ionizer could lead to tracheal tissue damage and biochemical changes, suggesting that high concentrations of NAIs may cause oxidative stress. Additionally, this phenomenon has not been found in NAIs generators of other brands. The laboratory subsequently reported that rats exposed to the same level of NAIs did not produce tissue damage but did change the indicators of reactive oxygen species. This response was also consistent with the lower degree of ozone exposure, which was considered to be the effect caused by the byproduct ozone (Sirota TV et al. 2008).

### Impact of NAIs on regulating emotion

It has been proposed that high-concentration NAIs exposure may reduce the severity of depression, psychological stress, and anxiety to improve well-being (Flory R et al. 2010). However, this conclusion is uncertain, as Perez V et al. found no correlations between air ions and emotion-related mental health (Perez V et al. 2013). The inconsistent findings may be due to confounding effects, such as air temperature, air humidity, air flow, electromagnetic field, and/or other unmeasured factors. Meta-analysis was conducted on five studies on negative ions and depression. The results showed that high-concentration NAIs exposure was significantly correlated with lower depression scores. The MD value in the high-level NAIs exposure group was 14.28 (95% CI: 12.93–15.62), and that in the low-level NAIs exposure group was 7.23 (95% CI: 2.62–11.83). Patients with seasonal or chronic depression can respond to higher levels of NAIs, but the effect of lower levels of NAIs is only observed in patients with seasonal depression.

Flory R et al. published a 5-year study to evaluate the efficacy of two active antidepressant therapies, white light and high-density NAIs, and two placebo therapies, dark red light and low-density NAIs, in seasonal affective disorder (SAD) (Flory R et al. 2010). Seventy-three female patients were included and exposed to one of the treatment methods in a controlled environment for 5 consecutive years in January of each year. A total of 12 consecutive cycles of treatment were carried out. Volunteers completed the seasonal pattern assessment questionnaire (SPAQ) before treatment and made a retrospective self-evaluation of the seasonal change patterns and degrees of sleep, social activities, mood, weight, appetite, and energy levels. After treatment, the treatment expectation questionnaire (TEQ), Structured Interview Guide for the Hamilton Depression Rating Scale-Seasonal Affective Disorder Version-Self Rating (SIGH-SAD-SR), and Beck Depression Inventory (BDI) were completed. The results revealed no significant difference in TEQ scores among the four groups. However, the SIGH-SAD-SR and BDI scores were positively correlated with NAIs treatment. Overall, the effect of white light therapy was higher than that of high-density anion therapy, both of which were higher than that of placebo therapy, but there was no significant difference in the effect of NAIs.

Bowers B et al. evaluated the continuous exposure of 40 SAD subjects to high-density or zero-density (placebo condition) NAIs for 18 days, 30 min, or 60 min a day (Bowers B et al. 2018). The results showed that high-density NAIs exposure was better than placebo in alleviating depression and atypical SAD symptoms. In the high-density anion group, 30 and 60 min of daily exposure could relieve depressive symptoms. In the high-density NAIs group, subjects with morning sleep were better treated than those with night sleep.

## Biological mechanism of NAIs action based on omics analysis

In recent years, high-throughput omics approaches have been powerful techniques to study large-scale dynamic molecular changes. Omics-based technologies allow for global and sensitive identification of molecular changes relevant for monitoring disease development (Mostafavi N et al. 2017). However, inconsistencies in research on the biological effects of NAIs warrant further mechanistic investigations in humans. Current research has revealed that the potential effects of NAIs on humans are mainly concentrated in the physiological and psychological fields, including the regulatory mechanism of the neurotransmitter 5-hydroxytryptamine (5-HT) and anti-inflammatory and antioxidant effects. However, the biological events induced by NAIs appear to be complex, and there is still an urgent need to study the molecular mechanisms of NAIs-induced biological effects on cytotoxicity in depth and breadth.

Omics is a powerful platform for screening internal exposure- and effect-related biological functions. Some epidemiological studies have adopted omics to investigate the relationship between environmental pollution and its effect on fundamental processes such as gene and microRNA expression, protein synthesis, enteric microorganisms, and metabolism (Chu JH et al. 2016; Fitch MN et al. 2020; Jardim MJ 2011; Wang W et al. 2018). Song X et al. (2022) integrated metabolomics, proteomics, and toxicology and suggested that PM_2.5_ exposure triggered the inhibition of the integrin signaling pathway and oxidative stress as intracellular ROS increased. Heavy metals and macrocyclic PAHs are important toxic components in PM_2.5_, triggering an increase in intracellular ROS and inducing the integrin signalling pathway, p53 signalling pathway, glycolysis, and fatty acid metabolism disorders. Based on their results, in the Dalian area, PM_2.5_ could interfere with glycolysis and the TCA cycle within carbohydrate metabolism throughout the year while only dramatically disturbing lipid metabolism in the winter. Espín-Pérez A et al. (2018) investigated the impact of short-term exposure (2 h) to air pollutants on the blood transcriptome and microRNA expression levels. PM_10_, PM_2.5_, CO, and CO_2_ exposures appear to be the most significant in terms of the number of hits (transcripts and microRNA) responding to exposure levels. The genes identified involve cancer-related pathways such as TP53, TGF-beta receptor signalling, p75, WNT-beta-catenin, and nonsense-mediated decay. MVN models revealed that hsa-miR-197-3p, hsa-miR-29a-3p, hsa-miR-15a-5p, hsa-miR-16-5p, and hsa-miR-92a-3p were significantly expressed in association with exposure.

**Fig. 3:**
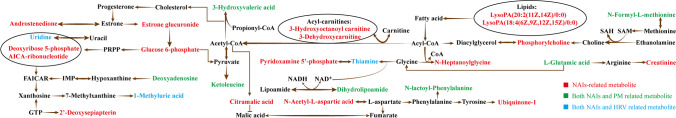
Schematic overview of the metabolic pathway associated with NAIs. Red: NAIs-related ones; green: common ones related to both NAIs and PM; blue: common ones related to both NAIs and HRV (this figure is part of Fig. 4 in the published paper by Liu S et al. and is used with permission.)

Many omics studies have investigated the mechanisms by which environmental pollutants interact with biology, but there are very few similar studies on NAIs. We retrieved only two omics studies of NAIs published by Liu S et al. in 2020 and 2022 (Liu S et al. 2020; Liu S et al. 2022). In 2020, they performed an untargeted metabolomics analysis of urine collected from an ionization air purifier intervention study in children to explore the molecular linkages between indoor NAIs, decreased PM, and the cardiorespiratory effect after purification via the meet-in-metabolite approach (MIMA) (Liu S et al. 2020). The results showed that twenty-eight metabolites were correlated with NAIs via linear mixed effect models. Scoring plots generated from partial least-squares discriminant analysis (PLS-DA) models present good separations of the metabolic profiles that characterized real and sham air purification. Thirteen of the twenty-eight metabolites were negatively associated with NAIs, while others were positively associated with them. In addition, forty-eight cardiorespiratory function-related compounds were further screened by two-way orthogonal PLS (O2PLS) models. Concerning NAIs-related metabolites, twenty-one are integrated with eleven respiratory function-related metabolites in the same pathways, which involve amino acid, lipid, glucose, pyrimidine, and purine metabolism, and benefit processes of energy production antioxidation and anti-inflammation. In addition, seventeen NAIs-related and eighteen HRV-related metabolites are integrated with uridine triphosphate (UTP) synthesis, the tricarboxylic acid (TCA) cycle, and amino acid and lipid metabolism. Overall, the metabolic network demonstrates the modes of energy production, antioxidation, anti-inflammation, and protecting nuclear integrity. Increased NAIs are related to ameliorated lung function with metabolic pathways similar to PM, mainly promoting energy production, anti-inflammation, and antioxidation reactions. Decreased PM ameliorated HRV with six main pathways, increasing energy production and anti-inflammation capacity, while increased NAIs deteriorated HRV with five main pathways, thereby lowering energy generation and antioxidation capacity (Fig. 3). Two years later, they published another repeated-measures panel study focusing on the associations of forest NAIs exposure with cardiac autonomic nervous function and the related metabolic linkages, and the results showed that forest NAIs were related to ameliorative changes in HRV indices, mainly through amino acid metabolism, anti-inflammation, and reduced inflammation, thereby promoting cardiac autonomic nervous function (Liu S et al. 2022).

**Fig. 4:**
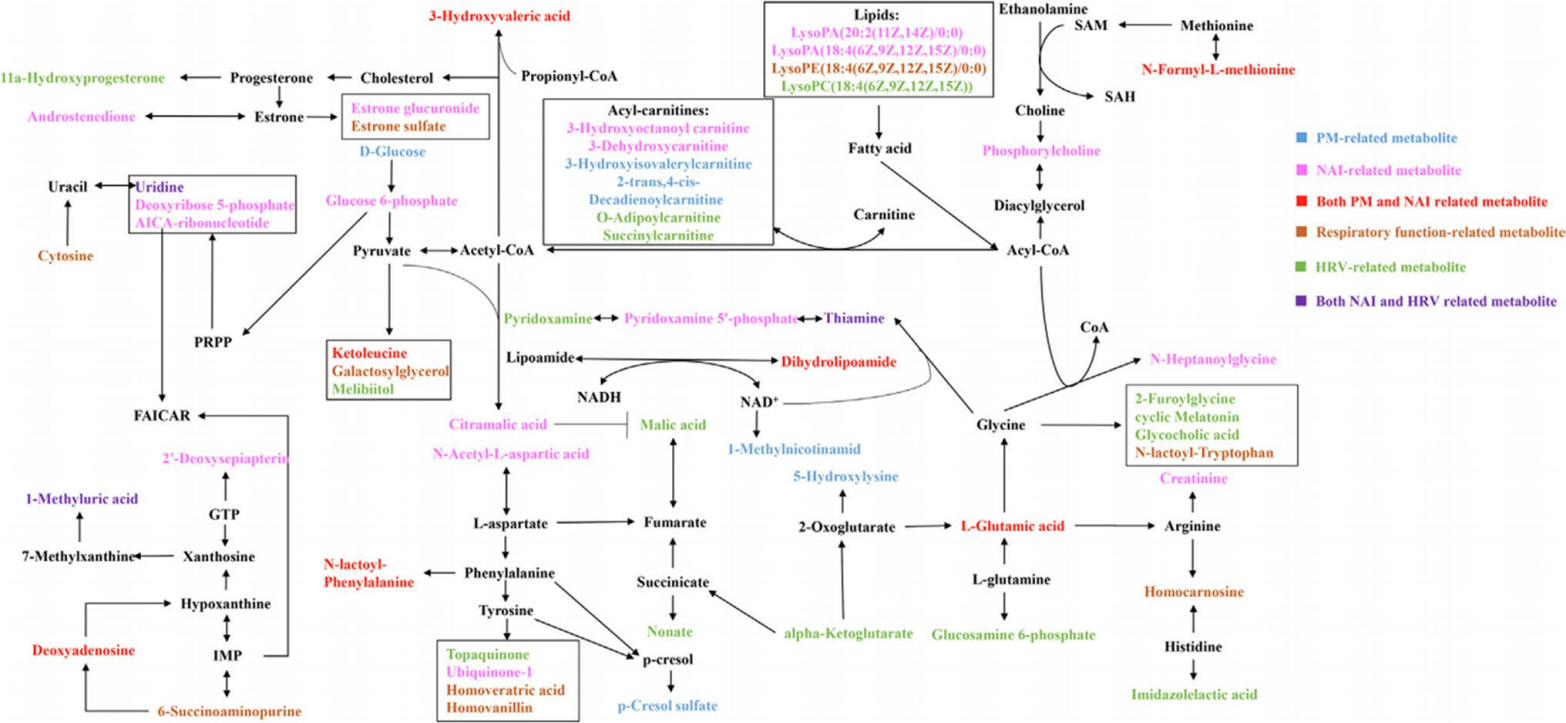
Note. Schematic overview of the metabolic pathway network. particulate matter (PM)-related metabolites, blue; negative air ion (NAI)-related ones, pink; respiratory function-related ones, brown; heart rate variability (HRV)-related ones, green; the common ones related to both PM and NAI, red, while the common ones related to both NAI and HRV, purple

## Mechanism implications and gaps in current knowledge

NAIs are known as “air vitamins.” The concentration of NAIs in the atmosphere is affected by the season, time, meteorological factors, air quality index, geographical location, forest vegetation, and other factors, while these factors also interact with each other. How their interaction affects the concentration of NAIs in the air and the mechanism of interaction need further analysis and summary.

NAIs have benefits on human health, such as the respiratory system, cardiovascular system, emotion, learning and memory, cognition, brain development, reproduction, growth and development, and anticancer effects. The diversity in the direction of gene and microRNA expression, protein synthesis, and metabolism toward disease indicates complex biological interactions between environmental exposure and omics signals. Therefore, integrated multiomics to identify biomarkers of NAIs health function is an effective research method. Some events reflect toxic risks, while others may indicate adaptation, damage repair, or both. The interpretation of changes in various molecular events in terms of disease risk remains a challenge, since the identified intermediate or ultimate biomarkers may be derived from different tissues and because the signals may relate to processes that have not previously been linked to diseases or observed in earlier stages during disease development. Furthermore, the same markers may show different biological functions in different diseases.

A systematic review of the above limited studies of NAIs exposure finds that most of the research in this arena is relatively old, and little new research exists on this topic in recent years. Furthermore, many studies suffer from various reporting and methodological deficiencies, posing the risk of study bias. These limitations include the sample size being too small, the experimental methods being different, a lack of sufficient data on the experimental design and results, and the temperature, humidity, and possible byproducts (i.e., the presence of an electric field, the production of ozone and other gaseous byproducts, noise, and light) being not appropriately controlled.

## Conclusions

In summary, our narrative review described the generation and temporal and spatial dynamic patterns of NAIs concentration as well as the relationship between NAIs concentration and health effects. Much of the research in this arena is relatively old, and very little new research on this topic has been pursued in recent years. Our conclusion is consistent with those of recent comprehensive reviews and meta-analyses of NAIs and their effects on health. Exposure to NAIs may benefit our health by changing amino acid metabolism, which mainly reflects increased anti-inflammation and reduced inflammation and antioxidation, promotes energy production, affects the expression of c-fos, or regulates 5-HT levels. Multiomics studies may provide a new perspective for research on the biological effects of NAIs.
